# Phyllospheric Microbial Composition and Diversity of the Tobacco Leaves Infected by *Didymella segeticola*

**DOI:** 10.3389/fmicb.2021.699699

**Published:** 2021-10-14

**Authors:** Yu Huang, Han-Cheng Wang, Liu-Ti Cai, Wenhong Li, Daiwei Pan, Ligang Xiang, Xiankun Su, Zhong Li, Muhammad Faheem Adil, Imran Haider Shamsi

**Affiliations:** ^1^Upland Flue-Cured Tobacco Quality and Ecology Key Laboratory of China Tobacco, Guizhou Academy of Tobacco Science, Guiyang, China; ^2^College of Agriculture, Guizhou University, Guiyang, China; ^3^Guizhou Institute of Plant Protection, Guizhou Academy of Agricultural Sciences, Guiyang, China; ^4^Faculty of Science, Wilfrid Laurier University, Waterloo, ON, Canada; ^5^College of Agriculture, Yangtze University, Jingzhou, China; ^6^Key Laboratory of Crop Germplasm Resource, Department of Agronomy, College of Agriculture and Biotechnology, Zhejiang University, Hangzhou, China

**Keywords:** microbial diversity, disease severity, *Didymella segeticola*, tobacco, high-throughput sequencing

## Abstract

A Myriad of biotic and abiotic factors inevitably affects the growth and production of tobacco (*Nicotiana tabacum* L.), which is a model crop and sought-after worldwide for its foliage. Among the various impacts the level of disease severity poses on plants, the influence on the dynamics of phyllospheric microbial diversity is of utmost importance. In China, recurring reports of a phyto-pathogen, *Didymella segeticola*, a causal agent of tobacco leaf spot, accentuate the need for its in-depth investigation. Here, a high-throughput sequencing technique, IonS5^TM^XL was employed to analyze tobacco leaves infected by *D. segeticola* at different disease severity levels, ranging from T1G (least disease index) to T4G (highest disease index), in an attempt to explore the composition and diversity of phyllospheric microbiota. In all healthy and diseased tobacco leaves, the most dominant fungal phylum was Ascomycota with a high prevalence of genus *Didymella*, followed by *Boeremia*, *Meyerozyma* and *Alternaria*, whereas in the case of bacterial phyla, Proteobacteria was prominent with *Pseudomonas* being a predominant genus, followed by *Pantoea*. The relative abundance of fungi, i.e., *Didymella* and *Boeremia* (Ascomycota) and bacteria, i.e., *Pseudomonas* and *Pantoea* (Proteobacteria) were higher in diseased groups compared to healthy groups. Healthy tissues exhibited relatively rich and diverse fungal communities in contrast with diseased groups. The infection of *D. segeticola* had a complex and significant effect on fungal as well as bacterial alpha diversity. FUNGuild analysis indicated that the relative abundance of pathotrophs and saprotrophs in diseased tissues proportionally increased with disease severity. PICRUSt analysis of diseased tissues indicated that the relative abundance of bacterial cell motility and membrane transport-related gene sequences elevated with an increase in disease severity from T1G to T3G and then tended to decrease at T4G. Conclusively, the current study shows the typical characteristics of the tobacco leaf microbiome and provides insights into the distinct microbiome shifts on tobacco leaves infected by *D*. *segeticola.*

## Introduction

Given its commercial importance, tobacco is cultivated extensively all across the globe and categorized as a non-food agricultural crop, mainly consumed as cigarettes, cigars, snuff, etc. (Liu et al., 2017). Its leaves are susceptible to damage by fungal, bacterial, and viral pathogens during growth and development. Morphological deformations, such as leaf spots induced by microflora, could result in heavy economical losses (Zhu, 2002). As a new taxon, *Didymella segeticola* was first reported by Chen et al. (2015), while its pathological characteristics were explained by Zhao et al. (2018) as being a causal agent of leaf spot on tea plants in China. Likewise, a study carried out by Guo et al. (2020) revealed that *D. segeticola* causes leaf spot disease in tobacco plants as well. Symptomatically, diseased tobacco leaves affected by this pathogen exhibit sandy beige-colored lesions with dark-brown edges, circular, elliptical, or irregular in shape, usually surrounded by yellow halos (Guo et al., 2020).

The phyllosphere or phylloplane (leaf surface) of a plant is an important habitat for many potentially beneficial, pathogenic, or antagonistic microbes (including bacteria, fungi, protists, and viruses), forming complex plant-microbial interactions and contributing greatly to plant health as well as productivity (Lindow and Brandl, 2003; Xin et al., 2016; Luo et al., 2019). However, the phyllospheric microbial community and composition are subjected to change by plant diseases (Luo et al., 2017; Zhang Z. et al., 2019). Tobacco powdery mildew infected by *Golovinomyces cichoracearum* was reported to reduce the leaf fungal community abundance and diversity (Huang et al., 2020); whereas a similar response was observed by Xiang et al. (2020c) during the investigation of tobacco brown spot caused by *Alternaria alternata*. Chen Q. L. et al. (2020) noticed a much higher abundance of *Rhizopus oryzae* in cured tobacco leaves infected by tobacco pole rot than that of healthy leaves. The change of phyllosphere microbe potentially helps to evaluate the effects of efficient disease management. The microbiome of tobacco leaves during the epidemic season of tobacco brown spot was significantly affected by the foliage application of dimetachlone, especially for the major pathogen *A. alternata* (Chen X. Y. L. et al., 2020). The biological control agent *Stenotrophomonas* displayed better performance in decreasing the disease index of wildfire than that of kasugamycin (Liu et al., 2021). Additionally, phyllospheric microbes were associated with a particular stage of disease severity for powdery mildew in *Euonymus japonicus* (Zhang Z. et al., 2019) and in *Cucurbita moschata* (Zhang et al., 2018). Efficient disease management of tobacco leaf spot caused by *D. segeticola* demands much consideration of the phyllospheric microbial composition and diversity of tobacco leaves, especially during disease epidemic season.

In recent years, high-throughput sequencing technologies have been used to investigate the characterizations of phyllosphere microbial community and composition, including target sequencing of the phylogenetic markers encoding 16S rRNA for bacteria and Internal Transcribed Spacer (ITS) for fungi (Zhang et al., 2016; Song et al., 2017). Notably, high sequencing flux, accuracy, and cost performance are a few of their advantages among many, and have been applied in multiple fields (Li et al., 2018; Mboowa et al., 2018; Speranskaya et al., 2018), and progressively utilized by researchers to study the interactions between host plants and their respective environments (Espenshade et al., 2019; Singh et al., 2019; Zhang Z. et al., 2019). Particularly in tobacco, these tools have been employed in the investigation of leaf microbiome for both healthy and diseased foliage infected with pole rot (Chen et al., 2019), brown spot (Liu et al., 2019; Liu et al., 2020; Xiang et al., 2020c), and powdery mildew (Huang et al., 2020). Disease severity is an important biotic factor to affect leaf microbiome, however, its influence upon the dynamics of phyllosphere microbial diversity is largely unexplored, especially for the newly reported tobacco leaf spot caused by *D. segeticola*. After the invasion of the pathogen, tobacco leaves normally show both symptomatic and asymptomatic parts, which could vary significantly with the advancement of disease severity. It is imperative to gather knowledge of the typical tobacco leaf microbiome characteristics and inspect the distinct microbiome shifts on tobacco leaves infected by this pathogen. In this study, tobacco leaves at different levels of disease severity were collected from a commercial field. Both fungal and bacterial compositions and diversity of tobacco leaves (symptomatic and asymptomatic parts) were analyzed by IonS5^TM^XL high-throughput sequencing technique, which led to some interesting findings. This study will provide a novel perspective on *D. segeticola* infection and its influence on the leaf microbiome at different leaf spot disease severity stages in tobacco.

## Materials and Methods

### Sampling Sites and Sampling Strategy

**FIGURE 1:**
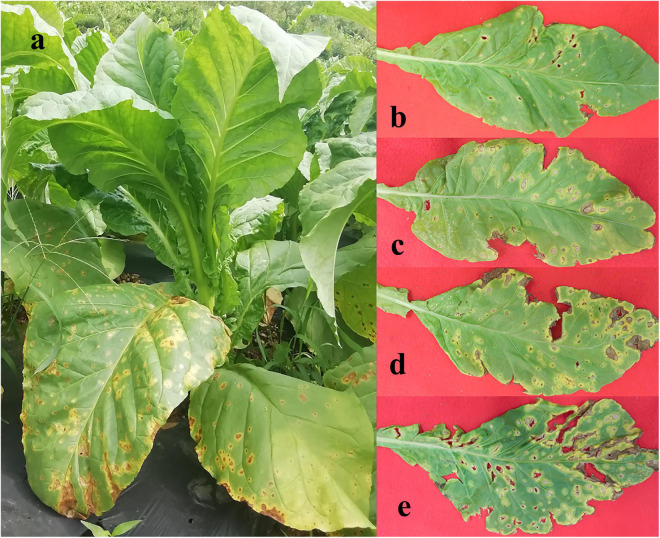
Whole tobacco plant **(a)** and leaves displaying disease severity at **(b)** T1G, **(c)** T2G, **(d)** T3G, and **(e)** T4G, respectively.

Leaf samples were obtained from tobacco plants (cultivar Yunyan 87) with symptoms of leaf spot disease caused by *D*. *segeticola* in June 2019, Zheng’an county (28.55° N,107.44°E), Guizhou Province, China. Tobacco leaves were randomly selected and classified into four different disease severity levels (T1G–T4G) based on the Chinese National Standard (GB/T 23222—2008). For T1G, T2G, T3G, and T4G groups, diseased lesion area ranged from 6 to 10%, 11 to 20%, 21 to 40%, and 41 to 100% of a leaf (Zhang et al., 2018), respectively. A total of 24 samples were collected and are illustrated in Figure 1. Three biological replicates from each group (same disease severity, different individual) were separated into two parts, with and without visible leaf spots, and labeled as diseased and healthy samples, respectively as presented in Table 1. After classification, leaf samples were immediately taken to the laboratory and stored at −80°C until further use.

**TABLE 1:** Sample information for both diseased and healthy tobacco leaves infected by *Didymella segeticola* at different disease severity.

**Disease severity**	**Proportion of lesion area every leaf**	**Disease grade**	**Diseased groups (sample)**		**Healthy groups (sample)**	
			**Fungi**	**Bacteria**	**Fungi**	**Bacteria**
T1G	6–10%	3	T1 (T11, T12, T13)	TX1 (TX11, TX12, TX13)	T1D (T1a, T1b, T1c)	TX1D (TX1a, TX1b, TX1c)
T2G	11–20%	5	T2 (T21, T22, T23)	TX2 (TX21, TX22, TX23)	T2D (T2a, T2b, T2c)	TX2D (TX2a, TX2b, TX2c)
T3G	21–40%	7	T3 (T31, T32, T33)	TX3 (TX31, TX32, TX33)	T3D (T3a, T3b, T3c)	TX3D (TX3a, TX3b, TX3c)
T4G	≥41%	9	T4 (T41, T42, T43)	TX4 (TX41, TX42, TX43)	T4D (T4a, T4b, T4c)	TX4D (TX4a, TX4b, TX4c)

*Samples and groups without “X” were used for ITS fungal sequencing, samples and groups with “X” were used for 16S bacterial sequencing.*

### DNA Extraction, PCR Amplification, and High-Throughput Sequencing

Each of the 0.5 g samples (stored at −80°C) was ground and FastDNA^®^ Spin kit for Soil (MP Biochemicals, Solon, OH, United States) was used according to the manufacturer’s protocol to extract the total DNA. Briefly, the powdered sample was added to Lysing Matrix E Tube, followed by the addition of sodium phosphate buffer (978 μL) and MT buffer (122 μL). After vortexing for 10–15 s, the samples were secured in FastPrep^®^ Instrument and processed for 30 s at 5.5 speed, then supernatants were transferred to a clean tube, and PPS reagent (250 μL) was added. To obtain the pellet, centrifugation (14,000 × *g* for 5 min) was performed, then the same amount of binding matrix suspension was added. To allow binding of DNA to the matrix, tubes were inverted to let silica matrix settle, then the supernatant was discarded. The binding matrix with the remaining amount of supernatant was re-suspended and approximately 600 μL of that mixture was transferred to a SPIN^TM^ Filter and centrifuged for 1 min (at 14,000 × *g*). SEWS-M (500 μL) was added to the SPIN^TM^ Filter and centrifuged again (at 14,000 × *g*) for 1 min. After decanting the flow-through, SPIN^TM^ Filter was replaced in a collection tube and centrifuged at 14,000 × *g* for 2 min to “dry” the matrix of residual SEWS-M wash solution. A fresh collection tube was employed to place the SPIN^TM^ Filter in, then left for air drying at room temperature (5 min). DNase/Pyrogen Free Water (DES; 50 μL) was added and the matrix on the filter membrane was gently vortexed to re-suspend the silica for efficient elution of DNA. Centrifugation at 14,000 × *g* for 1 min was performed to transfer eluted DNA to the collection tube. The total DNA concentration was adjusted to 30 ng μL^–1^ (Zhang et al., 2018), and the purity A260:A280 ratio was regulated within 1.8–2.2 by NanoDrop ND-2000 (Thermo Fisher Scientific, Waltham, MA, United States) before conducting PCR (Xiang et al., 2020a).

Following total DNA extraction, the fungal ITS region was amplified using the eukaryotic primers ITS1F primer (5′-CTTGGTCATTTAGAGGAAGTAA-3′) and ITS2R (5′-GCTGCGTTCTTCATCGATGC-3′) (Xiang et al., 2020a). To amplify the V3-V4 hypervariable regions of the 16S rRNA gene, 338F (5′-ACTCCTACGGGAGGCAGCAG-3′) and 806R (5′-GGACTACHVGGGTWTCTAAT-3′) primer pair were used (Xiang et al., 2020b). The PCR (amplification of the ITS region or V3-V4 region) was performed with a total volume of 20 μL (4 μL of 5 × FastPfu Buffer, 2 μL of 2.5 mM dNTPs, 0.8 μL of each primer, 0.4 μL of FastPfu Polymerase, and 1 μL DNA). The fungal PCR reaction was performed on a peqSTAR thermal cycler (PEQLAB Ltd., United Kingdom) with the following settings: 94°C for 5 min, followed by 35 cycles of 94°C for 1 min, 57°C for 1 min, and 72°C for 1 min, and finally 5 min at 72°C. The 16S rRNA gene reactions included the following steps: 94°C for 3 min, followed by 30 cycles of 94°C for 45 s, 55°C for 45 s and 72°C for 90 s, and finally at 72°C for 7 min. PCR products were checked by 2% agarose gel electrophoresis and purified with Gene JET (Thermo Fisher Scientific, Waltham, MA, United States), then sent for sequencing (250 bp paired-end sequencing) according to the standard protocol. The Ion S5 XL platform (Thermo Fisher Scientific, Waltham, MA, United States) was used for high-throughput sequencing at Novogene Bioinformatics Technology Co., Beijing, China.

### Data Processing

Single-end reads were generated (i.e., 400 bp/600 bp for fungi and 1,000 bp/1,200 bp for bacteria), followed by qualitative filtering and merging of raw reads by Cutadapt version 1.9.1.^1^ Afterward, the remaining unique reads were clustered into operational taxonomic units (OTUs) by UPARSE version 7.0.1001 software^2^ with a 97% similarity cutoff. Subsequently, the OTUs of each sample were annotated to different classifiers based on the UNITE version 7.2 fungal ITS and SILVA 132 rRNA database (Chen Q. L. et al., 2020; Xiang et al., 2020a). Finally, the estimates of diversity (Shannon and Simpson index), richness (Chao1 index), and beta diversity of microbial community were performed for different disease severity samples by Qiime software (Version 1.9.1). Generally, a higher Shannon and Simpson index represents a higher community diversity. In addition, higher Chao1 indices signify a higher community abundance (Zhang D. X. et al., 2019). Beta diversity on weighted and unweighted unifrac distance matrix was calculated by Qiime software (Version 1.9.1). Principal co-ordinate analysis (PCoA) was analyzed by WGCNA, stats, and ggplot2 package in R software (Version 1.9.1). Principal Component Analysis (PCA) was performed by using ade4 and ggplot2 packages in R software (Version 1.9.1). PCoA and PCA were used to determine the differences between community structures; samples with high similar communities tend to cluster together, on the contrary, samples become dispersed in the case of largely different communities (Zhang D. X. et al., 2019). Dilution curve, rank abundance curve, and species accumulation were drawn by R software (Version 2.15.3). The Venn diagrams were drawn by VennDiagram of software R (Version 3.0.3). FUNGuild database was used to analyze Fungal trophic mode (Nguyen et al., 2016). Bioinformatics software package PICRUSt was used to analyze bacterial OTUs for their metabolic functions according to Langille et al. (2013).

### Statistical Analysis

IBM SPSS Statistics 23 (IBM Corp., New York, United States of America) was used to analyze the data and to compare the differences of alpha-diversity indexes of fungal and bacterial communities (Mao et al., 2020). The mean values were compared and *P*-value at ≤ 0.05 was considered to be statistically significant.

## Results

### Quality of Total Fungal and Bacterial Sequence Data

**FIGURE 2:**
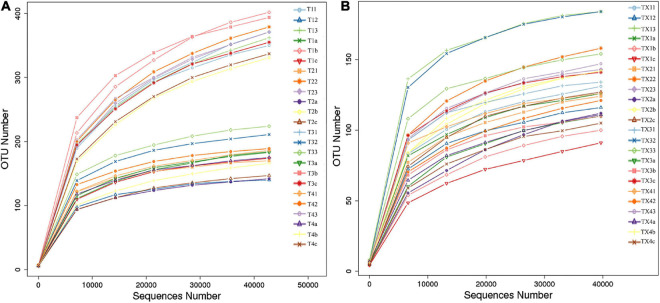
Rarefaction curves of fungal **(A)** and bacterial **(B)** OTUs across different tobacco leaf samples.

After quality control processing and de-noising, 1,713,851 fungal sequences were obtained from the 24 samples. The sequences were classified into 812 operational taxonomic units (OTUs) at a 97% similarity level. The fungal sequence of each sample was deposited in the SRA database under the accession number PRJNA714770. For bacteria, a total of 1,900,408 sequences were classified into 301 OTUs across the 24 samples. The bacterial sequence of each sample was deposited in the SRA database under the accession number PRJNA714984. When the number of sequences reached approximately 40,000, the rarefaction curves for all samples showed that they approached the plateau phase (Figure 2), suggesting that the sequence coverage accurately described the fungal and bacterial composition. For further analyses, the observed OTUs were used directly.

### Fungal and Bacterial Operational Taxonomic Unit Distribution and Diversity

**FIGURE 3:**
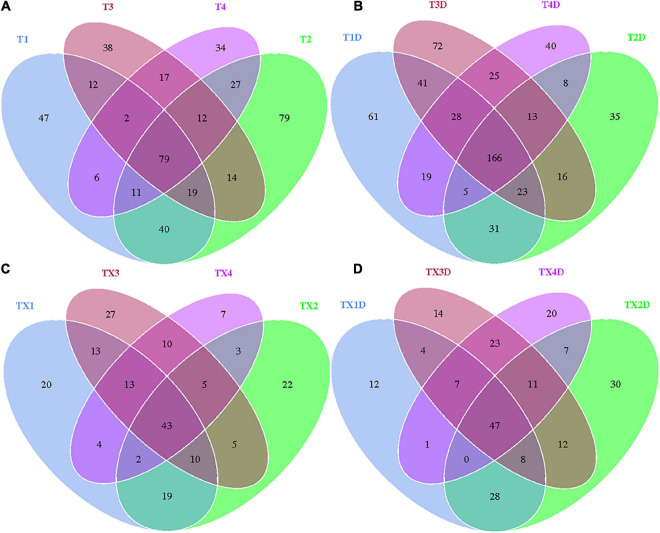
Venn diagram showing the number of fungal and bacterial OTUs detected in different groups at four different disease severity. Fungal Venn diagram in diseased **(A)** and healthy **(B)** groups at four different disease severities. Bacterial Venn diagram in diseased **(C)** and healthy **(D)** groups at four different disease severities. Numbers in the core section indicated shared OTUs for each group at four different disease severities. Numbers in the overlapping region indicated unique OTUs for two or three samples. Numbers in the non-overlapping regions indicated unique OTUs for the group.

A total of 812 fungal OTUs were obtained in Venn diagrams, including 437 belonging to the diseased group and 583 to the healthy group (Figure 3). The number of shared OTUs among the 4 diseased and healthy groups was 79 and 166, respectively. There were 47, 79, 38, and 34 OTUs found only in the T1, T2, T3, and T4 of the diseased group, respectively. Meanwhile, the healthy group T1D, T2D, T3D, and T4D only had 61, 35, 72, and 40 unique OTUs, respectively. The number of fungal OTUs in the healthy groups was more than those of the diseased groups. The Shannon index of diseased and healthy samples ranged from 3.31 ± 0.04 (mean ± SD) to 3.83 ± 0.07 and 4.45 ± 0.48 to 5.47 ± 0.37, respectively, whereas the Simpson index ranged from 0.73 ± 0.05 to 0.77 ± 0.05 for diseased, and 0.88 ± 0.01 to 0.95 ± 0.02 for healthy samples. The Chao1 index ranged from 153 ± 66 to 227 ± 101 and 192 ± 98 to 303 ± 103 for diseased and healthy samples, respectively. Correspondingly, the Shannon, Simpson and Chao1 indices indicated that the diversity and richness of the fungal community in the healthy groups were higher than those in the diseased groups (Table 2).

**TABLE 2:** Alpha-diversity indexes of fungal and bacterial community based on high-throughput sequencing in different samples.

**Disease severity groups**	**Shannon**	**Simpson**	**Chao1**	**Coverage**
**Fungi**				
T1	3.59 ± 0.50 cd	0.76 ± 0.05 *c*	208 ± 91 a	0.98
T2	3.83 ± 0.07 c	0.77 ± 0.05 bc	227 ± 101 a	0.99
T3	3.63 ± 0.63 cd	0.76 ± 0.05 c	156 ± 30 a	0.99
T4	3.31 ± 0.04 d	0.73 ± 0.05 c	153 ± 66 a	0.99
T1D	5.44 ± 0.86 a	0.95 ± 0.02 a	238 ± 132 a	0.99
T2D	4.45 ± 0.48 bc	0.88 ± 0.01 ab	285 ± 156 a	0.99
T3D	5.47 ± 0.37 a	0.93 ± 0.03 a	303 ± 103 a	0.99
T4D	4.84 ± 0.92 ab	0.91 ± 0.04 a	192 ± 98 a	0.99
**Bacteria**				
TX1	4.11 ± 0.82 a	0.89 ± 0.05 a	100 ± 16 a	0.99
TX2	3.21 ± 0.54 abc	0.80 ± 0.11 bc	99 ± 9 bc	0.99
TX3	4.06 ± 0.38 a	0.87 ± 0.07 ab	110 ± 6 ab	0.99
TX4	3.00 ± 0.69 bc	0.73 ± 0.13 ab	71 ± 6 a	0.99
TX1D	2.56 ± 0.11 c	0.68 ± 0.03 a	77 ± 7 c	0.99
TX2D	3.63 ± 0.77 ab	0.81 ± 0.08 bc	105 ± 11 abc	0.99
TX3D	2.89 ± 0.33 bc	0.70 ± 0.04 bc	92 ± 13 c	0.99
TX4D	2.52 ± 0.29 c	0.66 ± 0.08 c	99 ± 22 a	0.99

*Diversity and richness estimation of the fungal and bacterial sequencing libraries from the sequencing analysis. Shannon and Simpson were used to assess the community diversity, while Chao was used to evaluate the community richness. The values of mean ± SD of three samples are shown in the table, a P-value of < 0.05 was considered to be statistically significant. The same letter indicated that there were no differences between groups for fungi and bacteria, respectively, and different letters (a, b, c) indicated statistically significant differences.*

In the case of bacterial communities, a total of 301 OTUs were obtained, including 203 belonging to the diseased group and 224 to the healthy group. There were 43 and 47 shared OTUs found in four diseased and healthy groups, respectively. The unique OTU numbers in diseased groups from TX1 to TX4 were 20, 22, 27, and 7, respectively, while in the healthy group the numbers were 12, 30, 14, and 20, respectively. Accordingly, the Shannon index of diseased and healthy samples ranged from 3.00 ± 0.69 to 4.11 ± 0.82 and 2.52 ± 0.29 to 3.63 ± 0.77, respectively. Moreover, the Simpson index ranged from 0.73 ± 0.13 to 0.89 ± 0.05 for diseased and 0.66 ± 0.08 to 0.81 ± 0.08 for healthy samples. The Chao1 index ranged from 71 ± 6 to 110 ± 6 and 77 ± 7 to 105 ± 11 for diseased and healthy samples, respectively (Table 2). In general, the diversity of the bacterial community in the healthy groups was lower than in diseased groups, except for TX2D.

### Fungal and Bacterial Community Composition

#### Fungal Community Composition

**FIGURE 4:**
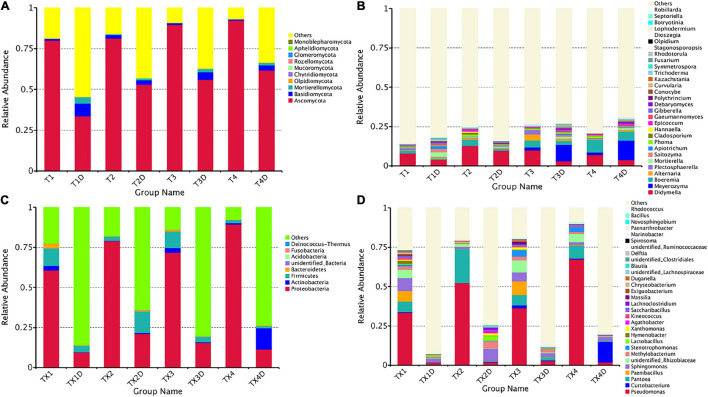
Microbial community composition of different groups at the phyla and genus levels. Fungal community composition of different groups at the phyla **(A)** and genus **(B)** levels. Bacterial community composition of different groups at the phyla **(C)** and genus **(D)** levels. Phyla making up less than 0.01 of total composition were classified as “Others”.

The ITS dataset showed that a total of 55.67% OTUs could be classified into the phyla of Ascomycota, Basidiomycota, Mortierellomycota, Olpidiomycota, Chytridiomycota, Mucoromycota, Rozellomycota, Glomeromycota, Aphelidiomycota, and Monoblepharomycota (Figure 4A). For diseased and healthy groups, the dominant fungal phylum was Ascomycota, followed by Basidiomycota and Mortierellomycota. Combining leaf tissue types, the relative abundance of Ascomycota in all diseased and healthy samples were 85.8 and 51.0%, Basidiomycota were 1.2 and 4.6%, and Mortierellomycota were 0.3 and 2.0%, respectively. Combining leaf tissue types and disease severity levels, the relative abundance of Ascomycota in diseased group T1, T2, T3, and T4 were 80.1, 81.3, 89.6, and 92.3%, respectively, while in healthy group T1D, T2D, T3D, and T4D, the percentage (%) was 33.5, 52.9, 56.0, and 61.6, respectively. In contrast, the relative abundance of Basidiomycota was 1.0, 2.1, 1.1, and 0.6% in group T1, T2, T3, and T4, respectively; while group T1D, T2D, T3D, and T4D represented a percentage of 7.9, 2.7, 4.6, and 3.3%, respectively. The relative abundance of Mortierellomycota was 0.2, 0.6, 0.1, and 0.3% (in group T1, T2, T3, and T4, respectively); whereas, percent values of 3.7, 1.1, 1.8, and 1.5 were obtained for group T1D, T2D, T3D, and T4D, respectively. Overall, the results infer that the contents of Ascomycota in diseased tissues were higher than in healthy tissues. Conversely, the contents of Basidiomycota and Mortierellomycota in diseased tissues were less than those in healthy tissues. In addition, with the increase in disease severity, the relative abundance of Ascomycota increased regardless of tissues being diseased or healthy.

**FIGURE 5:**
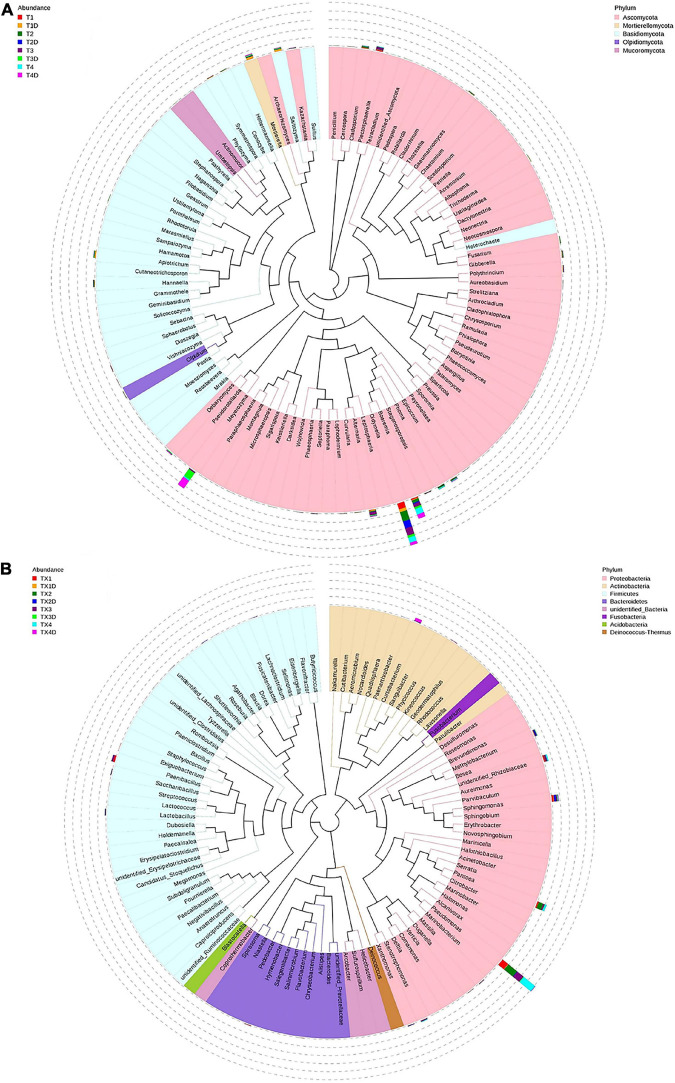
Maximum likelihood tree of the 100 most abundant fungal **(A)** and bacterial **(B)** genera in the eight group samples from tobacco leaves infected with *D. segeticola.* obtained by analysis of ITS rDNA and 16S rRNA pyrosequencing data. A color-coded bar plot shows the distribution of each fungal and bacterial genus in different groups.

At the genus level, the 30 most common fungal genera are shown in Figure 4B. Among those, the top 10 genera were *Didymella* (with a relative abundance of 7.3%), *Boeremia* (3.5%), *Alternaria* (1.1%), *Meyerozyma* (6.6%), *Plectosphaerella* (0.8%), *Mortierella* (0.8%), *Saitozyma* (0.6%), *Apiotrichum* (0.4%), *Phoma* (0.5%), and *Cladosporium* (0.5%) (Table 3). *Didymella* was the dominant genus inboth diseased and healthy groups. A maximum-likelihood tree of the 100 most abundant genera showed that the dominant fungi belonged to Ascomycota, followed by Basidiomycota and Mortierellomycota. *Didymella*, *Boeremia*, *Meyerozyma*, *Alternaria*, *Plectosphaerella*, *Phoma*, *Epicoccum*, and *Cladosporium* were the dominant fungal genera of Ascomycota. For the Basidiomycota, the dominant fungal genera were *Saitozyma*, *Apiotrichum*, and *Hannaella*. For the Mortierellomycota, *Mortierella* was the dominant fungal genus (Figure 5A).

**TABLE 3:** List of top 10 dominant taxa and their relative abundance in the fungal and bacterial community of the group.

**Fungal group**	***Didymella* (%)**	***Boeremia* (%)**	***Alternaria* (%)**	***Meyerozyma* (%)**	***Plectosphaerella* (%)**	***Mortierella* (%)**	***Saitozyma* (%)**	***Apiotrichum* (%)**	***Phoma* (%)**	***Cladosporium* (%)**
**T1**	8.0	1.7	0.5	< 0.1	1.3	0.2	0.1	0.0	0.2	0.7
**T2**	12.9	3.8	1.0	< 0.1	0.4	0.4	0.2	0.1	1.2	1.1
**T3**	10.0	4.4	3.8	1.8	3.0	0.1	0.1	0.0	0.4	0.2
**T4**	7.0	8.0	0.3	1.8	0.6	0.2	0.1	0.0	1.0	0.5
**T1D**	4.0	0.7	1.0	< 0.1	0.3	2.8	2.3	1.7	0.1	0.4
**T2D**	9.8	1.3	0.4	< 0.1	0.1	0.7	0.4	0.4	0.2	0.2
**T3D**	3.0	2.5	1.1	10.6	0.3	1.3	1.0	0.8	0.6	0.3
**T4D**	3.8	5.8	0.9	12.3	0.2	1.0	0.7	0.4	0.3	0.3

**Bacterial group**	***Pseudomonas* (%)**	***Pantoea* (%)**	***Paenibacillus* (%)**	***Curtobacterium* (%)**	***Sphingomonas* (%)**	***Methylobacterium* (%)**	***Stenotrophomonas* (%)**	***Lactobacillus* (%)**	***Hymenobacter* (%)**	***Xanthomonas* (%)**

**TX1**	33.3	6.4	6.8	0.8	8.3	5.3	2.1	1.2	0.6	0.8
**TX2**	52.3	21.4	0.4	0.1	1.2	1.7	1.0	0.0	0.3	0.4
**TX3**	36.4	6.7	8.5	1.7	5.7	7.6	2.5	4.2	0.1	1.3
**TX4**	67.5	8.1	0.4	0.6	1.7	5.4	1.3	2.9	0.1	0.1
**TX1D**	1.8	0.4	0.1	0.1	2.0	0.3	0.7	0.1	1.1	0.0
**TX2D**	2.0	0.4	< 0.1	0.3	7.9	0.3	4.6	0.3	3.0	1.5
**TX3D**	2.8	1.8	0.3	0.4	2.5	0.4	1.8	0.2	0.7	0.1
**TX4D**	1.8	0.5	0.2	13.2	2.4	0.2	0.4	0.3	0.1	0.0

The result related to the abundance of the top 10 fungal genera showed that *Didymella* in healthy groups was less compared to the diseased groups (Table 3). In diseased tissues, the relative abundance of *Didymella* increased with the progression of disease severity from T1G to T2G, and then decreased from T2G to T4G. Noticeably, healthy tissues exhibited the highest percentage of relative abundance for *Didymella* in T2D (9.8%), whereas for T1D, T3D, and T4D, it was found to be less (4.0%). *Boeremia* in healthy groups was less than those of the diseased groups, and with the increase of disease severity, the relative abundance of *Boeremia* increased. In diseased groups, the relative abundance of *Alternaria* increased proportionally with a disease severity level from T1G (0.5%) to T3G (3.8%), and then decreased in T4G (0.3%). In healthy groups, except for T2D (0.4%), the relative abundance of *Alternaria* was equal in T1D, T3D, and T4D. The relative abundance of *Meyerozyma* was higher in both diseased and healthy tissues of T3G and T4G. In contrast, *Mortierella*, *Saitozyma*, and *Apiotrichum* in healthy groups were higher than those of the diseased groups.

#### Bacterial Community Composition

The 16S dataset showed that a total of 94.68% OTUs could be classified in the Proteobacteria, Actinobacteria, Firmicutes, Bacteroidetes, Acidobacteria, Fusobacteria Deinococcus-Thermus, and unidentified-bacteria (Figure 4C). For diseased and healthy groups, the dominant bacterial phylum was Proteobacteria, followed by Firmicutes and Actinobacteria. Combining leaf tissue types, the relative abundance of Proteobacteria in all diseased and healthy samples were 75.2 and 14.4%, Firmicutes were 6.5 and 5.5%, and Actinobacteria were 1.8 and 3.7%, respectively. Combining tissue types and disease severity, the relative abundance of Proteobacteria in diseased group TX1, TX2, TX3, and TX4 were 60.7, 79.0, 71.8, and 89.4%, respectively, while in the healthy group TX1D, TX2D, TX3D, and TX4D were 9.6, 21.0, 15.4, and 11.5%, respectively. The relative abundance (%) of Firmicutes in diseased group TX1, TX2, TX3, and TX4 were 10.8, 2.6, 10.4, and 2.1, respectively; while in healthy group TX1D, TX2D, TX3D, and TX4D, were 3.9, 13.3, 3.4, and 1.3, respectively. Additionally, the relative abundance of Actinobacteria in diseased group TX1, TX2, TX3, and TX4 were 2.9, 0.3, 2.9, and 0.9%, respectively, while in healthy group TX1D, TX2D, TX3D, and TX4D were 0.2, 0.8, 0.6, and 13.2%, respectively.

In general, the results indicated that the contents of Proteobacteria and Firmicutes in diseased tissues were higher in comparison with healthy tissues. Relatively, the content of Actinobacteria in diseased tissues was less than those in healthy tissues. At the genus level, the 30 most common bacterial genera are shown in Figure 4D. The top 10 genera were *Pseudomonas* (24.7%), *Pantoea* (5.7%), *Paenibacillus* (2.4%), *Curtobacterium* (2.2%), *Sphingomonas* (4.0%), *Methylobacterium* (2.7%), *Stenotrophomonas* (1.8%), *Lactobacillus* (1.2%), *Hymenobacter* (0.8%), and *Xanthomonas* (0.5%) (Table 3). *Pseudomonas* and *Pantoea* were the dominant genera in the case of all diseased leaf groups, but not in the healthy groups. A maximum-likelihood tree of the 100 most abundant bacterial genera displayed that *Pseudomonas*, *Pantoea*, *Sphingomonas*, unidentified-*Rhizobiaceae*, and *Methylobacterium* were the dominant genera for the Proteobacteria, while *Paenibacillus* and *Curtobacterium* were the dominant bacterial genera for the Firmicutes and Actinobacteria, respectively (Figure 5B).

The abundance of the top 10 bacterial genera of all groups is presented in Table 3. The result revealed that *Pseudomonas*, *Pantoea*, *Paenibacillus*, and *Methylobacterium* in healthy groups were less than those of the diseased groups, indicating their prevalence in diseased tissues. In diseased groups, the relative abundance of *Pseudomonas, Pantoea, Paenibacillus*, and *Methylobacterium* ranged from 33.3 to 67.5%, 6.4 to 21.4%, 0.4 to 8.5%, and 1.7 to 7.6%, respectively, while in healthy groups, the abundance of *Pseudomonas, Pantoea, Paenibacillus*, and *Methylobacterium* was less than 3.0, 2, 0.3 and 0.4%, respectively.

### Spatial Distribution of Fungal and Bacterial Communities

**FIGURE 6:**
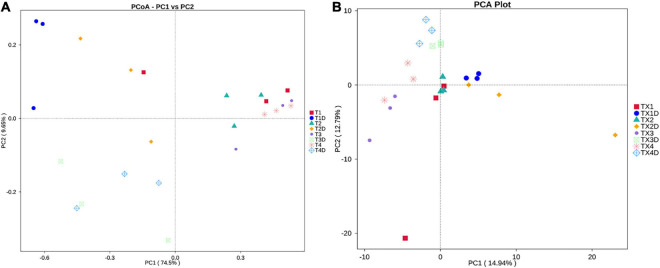
Principal Co-ordinate Analysis (PCoA) analysis of the fungal **(A)** and bacterial **(B)** communities in the different group samples.

PCoA plots were used to determine the spatial distribution of fungal and bacterial communities (Figure 6). In fungal communities, all diseased samples from four different disease severity levels tended to cluster together, except for one distinctive fungal community, which was from the diseased samples at the T1G disease severity (T12). On the other hand, the communities of healthy groups were clearly separated from each other (Figure 6A). In bacterial communities, all diseased and healthy samples were respectively closer to each other (Figure 6B). These results suggest that there were significant differences between healthy and diseased tobacco leaves apropos of fungal and bacterial communities, respectively, as affected by the *D. segeticola* infection.

### Fungal and Bacterial Functional Characteristics

**FIGURE 7:**
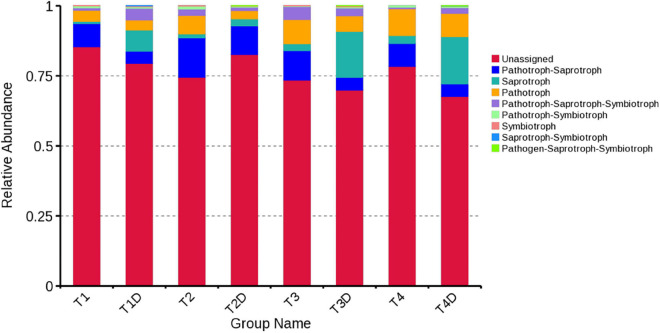
Relative abundance of fungal functional groups (guilds) based on OTU annotation table with disturbance frequency level.

**FIGURE 8:**
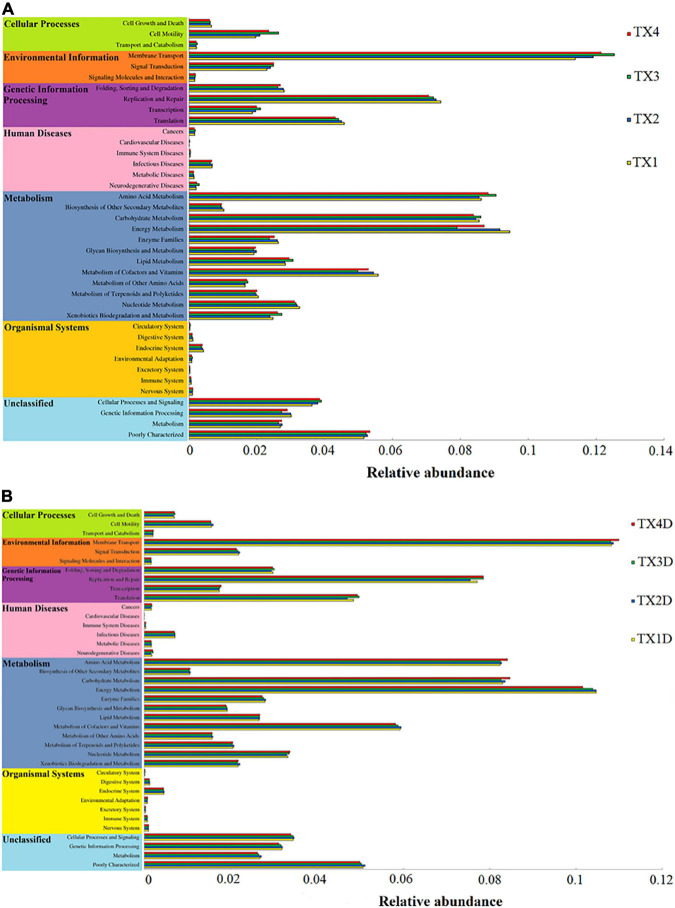
Variation of bacterial function categories in diseased **(A)** and healthy **(B)** groups at different disease severity analyzed by PICRUSt.

The fungal OTUs were categorized into nine groups according to their trophic mode. Pathotroph-saprotroph, pathotroph, and saprotroph were the dominant trophic modes in the composition of the tobacco leaves (Figure 7). Generally, the abundance of pathotroph-saprotroph and pathotroph in diseased groups was found to be higher than those of healthy groups, while this was not the case for saprotroph abundance. In diseased groups, the relative abundance of pathotroph and saprotroph increased along with the increase in disease severity from T1G to T4G. For the variation of bacterial functional categories, the gene sequence in diseased and healthy groups (as shown in Figures 8A,B), were mostly involved in metabolism functions, followed by genetic information processing and environmental information processing. Under the category of metabolism functions, amino acid metabolism, carbohydrate metabolism, and energy metabolism were highly enriched in diseased samples, whereas in the case of healthy samples, in addition to the aforementioned categories, the metabolism of cofactor and vitamins were also enriched significantly with a declining trend from TX1D to TX4D. For environmental information, membrane transport activity in diseased leaves followed the pattern: TX3 > TX4 > TX2 > TX1, while in the context of healthy ones, TX4D was observed to be enriched more than the other three categories. The terms, i.e., translation, replication, and repair, under the category of genetic information processing displayed a gradual decline from TX1 to TX4 in diseased samples, albeit the healthy samples exhibited higher enrichment for TX3D and TX4D than TX1D or TX2D. The relative abundance of gene sequences was not corrected with increased disease severity.

## Discussion

In the present study, the ITS region of rDNA and V3-V4 hypervariable regions of the 16S rRNA were amplified to detect the dynamics of microbial diversity to different disease severity levels of *D. segeticola* infection, which manifests as leaf spots in tobacco. Tobacco leaves were separated into healthy and diseased categories for analysis; the relative abundance of fungi, i.e., *Didymella* and *Boeremia* (Ascomycota) and bacteria, i.e., *Pseudomonas* and *Pantoea* (Proteobacteria) were higher in diseased groups compared to healthy groups. Healthy tissues exhibited quite rich and diverse fungal communities compared with those of diseased groups. The analysis of Venn indicated that there were more fungal OTUs in the healthy groups than in diseased groups, which is in line with a previous study conducted by Rosenzweig et al. (2012) demonstrating a higher number of OTUs in the rhizosphere of healthy soil compared to diseased soil. Moreover, the number of fungal and bacterial OTUs was subject to change with increase in disease severity. In healthy groups, the dynamics of fungal OTUs were opposite to the bacterial OTUs with the disease severity advancement, which might be related to the factor of leaf nutrition leading toward the competitive relationship between microorganisms.

In our study, results suggested a higher fungal alpha diversity in healthy groups than diseased groups, which is in agreement with previous studies (Shang et al., 2016; Zhang Z. et al., 2019). The analysis revealed that the fungal alpha diversity of diseased groups decreased with an increased disease severity level from T2G to T4G. Research conducted by Manching et al. (2014) validates our results, where they reported an inverse correlation between alpha diversity and severity of southern leaf blight disease under immense disease pressure. The trend of fungal alpha diversity change was the same as the relative abundance of *Didymella* with increased disease severity in diseased groups, attributing the changes in fungal alpha diversity to the pathogen invasion. The ITS dataset showed that the fungal community of the tobacco phyllosphere was altered by disease severity. In both healthy and diseased groups, Ascomycota was the most dominant phylum, where its relative abundance increased gradually with disease severity level, a trend similar to which has been reported by Zhang et al. (2018) while working with pumpkin leaves affected by powdery mildew. At the genus level, fungal OTUs were mostly dominated by *Didymella*, followed by *Boeremia* and *Alternaria*. *Didymella* was the most dominant genus in all diseased groups and some earlier reports are consistent with this finding (Chen et al., 2019; Zhang Z. et al., 2019). Our study indicated that the highest relative abundance of this pathogen was observed in T2G rather than T4G, which contradicts with the study of Zhang et al. (2018) who indicated that the abundance of the pathogen genus *Podosphaera* was positively correlated with a spike in pumpkin powdery mildew disease severity. We corroborate that the parasitic ability of *Didymella* is inferior to *Podosphaera*, which might benefit the growth of other microorganisms post-*Didymella* genus invasion. Therefore, the relative abundance of *Alternaria* rose with an increase in disease severity from T1G to T3G and then decreased at T4G in diseased groups. It could be deduced that genus *Alternaria* could benefit from the disease pressure caused by *Didymella*. The relative abundance of *Boeremia* was found to be positively correlated with disease severity in diseased groups. Previous studies have reported the *Boeremia* genus as an endophytic fungus and a pathogen of tobacco (Ibrahim et al., 2017). It may be explained with the disease severity increase from T2G to T4G in the diseased group, where the relative abundance of *Didymella* decreased, while the relative abundance of *Boeremia* increased.

Tobacco phyllosphere fungal composition in our study was mostly identified as Pathotroph-saprotroph, saprotroph, and pathotroph. With disease severity increase, the trend of change in the relative abundance of pathotroph was the same as those of saprotroph in both diseased and healthy groups. This result indicated that there were many fungi with potential pathogenic characteristics on tobacco phyllosphere, as well as that most of those fungi are saprotrophs, which include genera whose species are well-known tobacco pathogens, for instance, *Alternaria alternata* (Wang et al., 2016), *Didymella segeticola* (Guo et al., 2020), *Phoma sorghina*, and *Phoma omnivirens* causing leaf spot on tobacco (Yuan et al., 2016; Jiang et al., 2018), as well as *Cladosporium cladosporioides* causing seed disease in tobacco (Wang et al., 2014). In general, most of these pathogens that infect tobacco leaves could grow both pathogenically inside the leaves or saprophytically on leaf surfaces. However, the abundance of microorganisms could be affected by many factors, such as sampling time, sampling place and plant species, etc. The 16S dataset showed that the bacterial alpha diversity became complicated with increased disease severity, owing to the tobacco leaf spot caused by *D*. *segeticola*, which may influence other fungal communities. The lowest bacterial alpha diversity under T4G in diseased groups may be attributed to the high disease pressure, inhibiting bacterial growth. Except for the T2G, the bacterial community diversity in the healthy groups was lower than those in the diseased groups. This result resonates with a previous study, where the bacterial diversity in the stem of black shank tobacco plants was found to be higher than that of healthy stems (Xiang et al., 2020b). The bacterial community diversity of TX2D was higher than the TX2, owing to the fact that T2G had a large *Pantoea* and *Didymella* population, consequently inhibiting the growth of other bacteria in the diseased part.

Regarding the bacterial community, Proteobacteria was the most abundant phylum in all groups and has been reported as a dominant bacterial taxon in many phyllospheric communities and in tobacco stems infected by fungal diseases (Liu et al., 2020; Xiang et al., 2020b). At the genus level, *Pseudomonas* and *Pantoea* were the dominant genera in diseased groups, which is in correspondence with an earlier report (Liu et al., 2020). In this study, the pattern of change in the relative abundance of *Pseudomonas* and *Pantoea* was the same as the *Didymella* from disease severity T1G to T3G in diseased groups. There may be a synergistic or antagonistic relationship between *Didymella* and two bacterial genera of *Pseudomonas* and *Pantoea*. Genus *Pseudomonas* potentially causes diseases in tobacco, such as wildfire (caused by Pseudomonas syringae pv. tabaci), and angular leaf spot (caused by *Pseudomonas syringae* pv. *angula*), and while looking at the bigger picture, *D. segeticola* and *Pseudomonas syringae* pv. *tabaci* could work hand-in-hand for their mutual benefit, as tobacco leaf spot caused by *D. segeticola* (Guo et al., 2020) might mix with tobacco wildfire or angular tobacco leaf spot (Cole, 1960; Zhu, 2002; Guo et al., 2017). More work should be conducted to verify this hypothesis. However, some studies reported that *Pantoea* spp. elicited a hypersensitive reaction in tobacco leaves (Mondal et al., 2011; Wang, 2018). The relative abundance of *Pantoea* in diseased groups were found higher than those of healthy groups, combined with the trend of change in the relative abundance of *Pantoea* being the same as the *Didymella* from disease severity T1G to T3G in diseased groups, supporting the evidence that the *Pantoea* could produce a hypersensitive reaction to prevent attack by pathogens. Results from PICRUSt analysis indicated that the metabolism of energy, cofactors, and vitamins declined with an increase in disease severity level from T1G to T3G in diseased groups, which may be associated with the factor of disease pressure. However, cell motility and membrane transport increased with disease severity level from T1G to T3G in diseased groups, the trend of change was the same as the relative abundance of phototrophs with increased disease severity from T1G to T3G. The results indicate that many microorganisms could provide corresponding material for pathogen growth. The reason why the cell motility and membrane transport were decreased at T4G may be attributed to some of the compounds produced by microorganisms which inhibit biofilm development in the diseased group. Morales et al. (2013) reported bacterial molecules such as *Pseudomonas aeruginosa* produced phenazines or compounds with similar activities inhibiting biofilm development.

## Conclusion

The obtained data from this study helps to infer that the composition and diversity of phyllospheric microbiota differed markedly on *D. segeticola*-infected tobacco leaves at different disease severity levels, particularly in the case of the fungal community. Noticeably, in all tobacco leaf samples, the most dominant fungal phylum was Ascomycota with a high prevalence of genus *Didymella*, co-existing with bacterial phylum Proteobacteria with predominant genus *Pseudomonas*, however, their relative abundance was found to be higher in diseased groups compared to healthy counterparts. Conclusively, healthy tissues exhibited quite rich and diverse fungal communities in contrast with diseased groups. The infection of *D. segeticola* had a significant but varying effect on bacterial alpha-diversity. The relative abundance of pathotrophs and saprotrophs proportionally increased in diseased tissues with the progression of disease severity.

## Data Availability Statement

The datasets presented in this study can be found in online repositories. The names of the repository/repositories and accession number(s) can be found in the article/supplementary material.

## Author Contributions

YH, H-CW, and IS contributed to conception and design of the study. YH organized the database and performed the statistical analysis. YH and H-CW wrote the first draft of the manuscript. IS and ZL wrote sections of the manuscript. All authors contributed to manuscript revision, read, and approved the submitted version.

## Conflict of Interest

The authors declare that this study received funding from Guizhou Tobacco Company Project. The funder was not involved in the study design, collection, analysis, interpretation of data, the writing of this article or the decision to submit it for publication.

## Publisher’s Note

All claims expressed in this article are solely those of the authors and do not necessarily represent those of their affiliated organizations, or those of the publisher, the editors and the reviewers. Any product that may be evaluated in this article, or claim that may be made by its manufacturer, is not guaranteed or endorsed by the publisher.

## Footnotes

1
http://cutadapt.readthedocs.io/en/stable/

2
http://www.drive5.com/uparse/
